# Electrospun Hydrophobic Polyaniline/Silk Fibroin Electrochromic Nanofibers with Low Electrical Resistance

**DOI:** 10.3390/polym12092102

**Published:** 2020-09-16

**Authors:** Chun-Yu Chen, Szu Ying Huang, Hung-Yu Wan, Yi-Ting Chen, Sheng-Ka Yu, Hsuan-Chen Wu, Ta-I Yang

**Affiliations:** 1 Department of Chemical Engineering, Chung-Yuan Christian University, Taoyuan 32023, Taiwan; j369369369369@gmail.com (C.-Y.C.); sallyhuang0704@gmail.com (S.Y.H.); wanwan850109@gmail.com (H.-Y.W.); tina860930@gmail.com (Y.-T.C.); internet6112@gmail.com (S.-K.Y.); 2 Department of Biochemical Science and Technology, National Taiwan University, Taipei 10617, Taiwan

**Keywords:** electrospinning, electroactive nanofiber, silk fibroin

## Abstract

Electronic textiles (E-textiles) have been an area of intense industrial and academic research for years due to their advanced applications. Thus, the goal of this study was to develop highly conductive silk fibroin electrochromic nanofibers for use in E-textiles. The silk nanofibers were prepared by an electrospinning technique, and the conductive polyaniline (PANI) was added to impart the electrical conductivity and electroactive property to the resultant electrospun silk composite nanofibers. The experimental results showed that tuning the electrospinning procedure could control the morphology of the composite nanofibers, thus altering their mechanical properties and surface wettability. Furthermore, the developed PANI/silk composite fibers possess electroactive and electrochromic properties, such as adjusting the applied voltage. The developed strategy demonstrated the feasibility of incorporating not only electrical functionality but also electroactivity into sustainable silk nanofibers using electrospinning technique.

## 1. Introduction

Electronic textiles (E-textiles) have been an area of intense industrial and academic research for years since they are considered critical in a broad range of wearable applications including physiochemical sensors, thermoelectric fibers/fabrics, artificial muscles, medical textiles, protective clothing, touch screen displays, and textile-based energy devices and systems [1,2,3]. Thus, conductive fibers/fabrics, which are the key component of E-textiles, are in great demand. Many efforts have been devoted to developing conductive fibers from traditional textile materials such as cotton, silk, polyester, and nylon [4]. Metallizing fibers with metallic nickel, copper, or silver is the simplest way to obtain conductive fibers. However, the disadvantage of metals is that they have low elasticity and strength so that metal-coated fibers and fabrics can corrode and crack after time [5].

Recently, intrinsically conductive polymers (ICP), including polyaniline (PANI), polypyrrole (PPy) and poly(3,4-ethylenedioxythiophene) (PEDOT), were utilized to develop conductive fibers because they are lightweight, cost effective, and environmentally stable compared to inorganic metals [3]. Specifically, PANI has attracted much interest worldwide because not only is its thermal and chemical stability superior to other ICPs, but also it can be processed by solution-processing techniques [3,6]. Researchers have successfully utilized PANI to obtain conductive poly(vinylidene fluoride), polyester, nylon, polyimide, and aramid fibers [7,8,9,10,11].

However, there is an increasing demand for biodegradable E-textiles, which can relieve the environmentally critical problem of discarded electronic waste without leaving a permanent mark on our environment [12]. One of the beginning efforts to develop biodegradable ICP composite fibers was by incorporating ICPs with synthetic biodegradable polymers, such as polylactide (PLA), polycaprolactone (PCL), and polyurethane (PU) [13]. Furthermore, with strong growth expected in electronic textiles, it is essential that conductive elements meet not only environmental considerations but also sustainable resources, indicating that they can be continuously replenished without any shortage. Therefore, the naturally derived silk fiber is emerging as a promising material to develop conductive fibers due to its biodegradability and sustainability. Researchers have demonstrated the success of utilizing pristine silk fibers to develop conductive silk fiber for applications in E-textiles including conducting wires, ammonia/acetaldehyde sensors, electronic elements, and thermoelectric modules [14,15,16,17]. However, pristine silks collected from silkworms can exhibit high batch-to-batch variation of fiber structure and also lack flexibility to design and thus achieve desired fiber morphologies for designated applications. 

In this study, we demonstrated the feasibility of utilizing electrospinning technique to fabricate conductive PANI/silk nanofibers. Electrospinning technique is well known for its capability to develop nanofibrous materials with desired microstructures [18,19]. We can control the electrospun PANI/silk composite nanofibers with various microstructures by tuning the electrospinning parameters including applied electric field, solution flow rate, and distance between the needle and collector. The mechanical properties, surface wettability, and electrochemical-responsive behaviors of the electrospun PANI/silk nanofibers were systematically studied. The reported experiment results could provide a new avenue for developing biodegradable, sustainable, and conductive nanofibers for use in E-textiles.

## 2. Experimental

### 2.1. Materials 

Sodium carbonate (99%, Showa, Tokyo, Japan), anhydrous lithium bromide (99%, Showa, Tokyo, Japan), ammonium persulfate (98%, Showa, Tokyo, Japan), aniline monomer (99%, Sigma, St. Louis, MO, USA), hydrazine hydrate (50–60%, Sigma, St. Louis, MO, USA), and formic acid (98%, Fluka, Muskegon, MI, USA) were used as received without further purification. *Bombyx mori* silkworm cocoon was from a local supplier based in Taiwan (Quanming Silk Farm, Miaoli, Taiwan). 

### 2.2. Preparation of Degummed Silk Fibroin

Native silkworm (*Bombyx mori*) silk is composed of silk fibroin protein coated with sericin proteins. The silk fibroin for preparing electrospun PANI composite fibers was prepared according to published procedure [20]. The sericin was removed by a “degumming” process, which is boiling the silkworm cocoons in 0.02 M sodium carbonate solution for 30 min. Subsequently, the dried degummed silk fibers were dissolved in 9.3 M lithium bromide solution for 4 h at 60 °C. The resultant fibroin solution was dialyzed using a dialysis cassette with cutoff molecular weight of 3500 for 72 h, and then lyophilized for long-term storage.

### 2.3. PANI Synthesis

PANIs were synthesized by polymerizing aniline monomer with ammonium persulfate as the oxidizing agent. The aniline (10 g) was dissolved in 200 mL of 1 *N* hydrochloric acid solution and then cooled the solution to 0 °C for 30 min. Subsequently, a solution of 6.13 g ammonium persulfate in 20 mL deionized water was slowly added into the aniline solution. The resulting mixture was allowed to react at 0 °C for four hours. The synthesized product (emeraldine salt state of PANI) was collected by centrifugation and washed with 1 *N* ammonium hydroxide solution, resulting in emeraldine base of PANI. All the results of PANI’s characterization are included in Supplementary Materials.

### 2.4. Preparation of PANI/Silk Composite Nanofibers 

PANI/silk composite nanofibers were prepared by using an electrospinning apparatus (FES-COD, Falco Tech Enterprise Co. Ltd., New Taipei City, Taiwan). The required amounts of lyophilized silk fibroin and PANI for electrospinning process were dissolved in formic acid, as summarized in Table 1. Subsequently, the resulting solution was ejected through a syringe with a needle size of gauge 20. A high-voltage power supplier provided the required voltage to the needle and the tip-target distance for electrospinning maintained at 12 cm. The feeding rate (0.1–0.8 mL/h) and applied voltage (15–30 kV) for electrospinning were systematically tuned to obtain PANI/silk composites with fiber morphology. The ambient temperature and relative humidity for electrospinning were controlled in the range of 25–28 °C and 60–70%, respectively. The obtained electrospun mats were stored in the vacuum oven at room temperature.

### 2.5. Characterization

#### 2.5.1. Fourier Transform Infrared (FTIR) Measurement

A JASCO FTIR-4100 spectrometer with an ATR function (JASCO International Co. Ltd., Tokyo, Japan) was utilized to obtain chemical structure of the synthesized PANI and electrospun PANI/silk samples. FTIR analysis was carried out at room temperature on solid PANI or PANI/silk samples at a resolution of 4.0 cm^−1^ over a range of 4000–800 cm^−1^.

#### 2.5.2. Scanning Electron Microscopy (SEM) of Electrospun Nanofibers 

Dry PANI/silk composite nanofibers were put on SEM specimen stubs, coated with palladium for two minutes, and their morphologies were observed using a Hitachi S-2300 scanning electron microscope (Hitachi, Tokyo, Japan) with an operating accelerating voltage of 10 kV.

#### 2.5.3. Tensile Tests 

The mechanical properties of the electrospun PANI/silk composite nanofibers’ mats were tested by a universal testing machine (FAL-508M1F, Falco Tech Enterprise Co. Ltd., Taipei, Taiwan). Rectangular electrospun mats, approximately 10 mm wide and 40 mm in length, were prepared and then mounted on the testing machine. The uniaxial tensile test was performed at a pulling rate of 50 mm/ min at room temperature until samples fractured. The machine-recorded data were used to obtain the tensile stress–strain relationship of the specimens and then the tensile strength and elongation strain of the prepared electrospun PANI/silk mats were determined. Five specimens of each sample were tested to obtain the mean values and standard deviation.

#### 2.5.4. UV-Visible Analysis

UV-visible spectra of PANI were obtained using a JASCO V-750 UV-VIS spectrophotometer (JASCO International Co. Ltd., Tokyo, Japan). The as-prepared emeraldine base (EB) of PANI was dissolved in n-methyl-2-pyrrolidone (NMP) for measurement at room temperature. Furthermore, hydrazine could completely transform as-prepared PANI from EB form to leucoemeraldine base (LEB) form. The procedure was as follows:

The as-prepared emeraldine base (EB) of PANI (0.4 g) was dispersed in a mixed solution of 4 mL hydrazine hydrate and 50 mL 1.0 M ammonium hydroxide and then stirred for 10 h. Subsequently, the solution was filtered and washed with distilled water. The obtained solid product was the PANI with LEB form. Therefore, the completely reduced PANI (LEB form) could dissolved in NMP and then was added to ammonium persulfate for in situ monitoring of the sequential oxidation process of PANI with LEB form by UV-VIS spectrophotometer. 

#### 2.5.5. Electrochemical Cyclic Voltammetry (CV) Study 

The redox behavior of the prepared PANI/silk composite nanofibers was investigated using CV measurement (Autolab PGSTAT204, Metrohm, Herisau, Switzerland). The composite fibers were attached on an indium tin oxide glass serving as a working electrode. The CV measurement was performed in 1.0 M sulfuric acid solution. The testing potentials ranged from −0.2 to 1.0 V at a scan rate of 50 mV·s^−1^ using silver/silver chloride reference electrode and platinum counter electrode.

#### 2.5.6. Electrical Conductivity Measurement

The electrical conductivity of the prepared PANI/silk nanofiber mats was determined by the four-point probes method (LRS4-TG, KeithLink Technology Co. Ltd., New Taipei City, Taiwan). The composite mats were doped with hydrochloric acid to improve their conductivity. 

#### 2.5.7. Porosity Measurements

The porosity (ε) of the electrospun PANI/silk composite nanofibers’ mats was evaluated at room temperature using the liquid intrusion method [21]. Accordingly, the electrospun mats were weighed (*m*_1_) and then immersed in ethanol to allow the liquid to penetrate into the void volume. The composite fibers were taken out, blotted with a Kimwipe, and reweighed (*m*_2_) to determine the mass of ethanol present within the composite fibers. The porosity (ε) was calculated as

(1) $$\varepsilon =\frac{V_{EtOH}}{V_{EtOH}+V_{mat}}\left(V_{EtOH}=\frac{m_{2}-m_{1}}{\rho_{EtOH}},\;\;V_{mat}=\frac{m_{1}}{\rho_{mat}}\right),$$

where *m*_1_ is the mass of the dry electrospun mats and *m*_2_ is the mass of electrospun mat with ethanol, respectively. *V_EtOH_* is the volume of intruded ethanol and *V_mat_* is the volume of the composite mats, respectively. The density of ethanol (*ρ_ETOH_*) was 0.8 g/cm^3^ while the density of composite mats was 1.3 g/cm^3^. Note: Silk and PANI have similar densities, of 1.3 g/cm^3^ [22,23].

## 3. Results and Discussion

### 3.1. Electrospinning PANI/Silk Composite Nanofibers 

We first studied the electrospinning conditions for fabricating silk fibroin (SF) nanofibers before preparing PANI/silk composite nanofibers. In principle, a number of electrospinning parameters including polymer concentration, solution feeding rate, applied voltage, and distance between electrodes could influence spinnability and the resulting fiber morphology structures [19]. Most importantly, the polymer concentration is crucial for electrospinning, which leads to morphologies ranging from beads to fibers [24]. The SF concentration suitable for achieving fibrous morphology was chosen as 10 wt.% after several preliminary tests. Subsequently, we fine-tuned the solution feeding rate and electric field to obtain silk fibroin nanofibers. 

**Figure 1:**
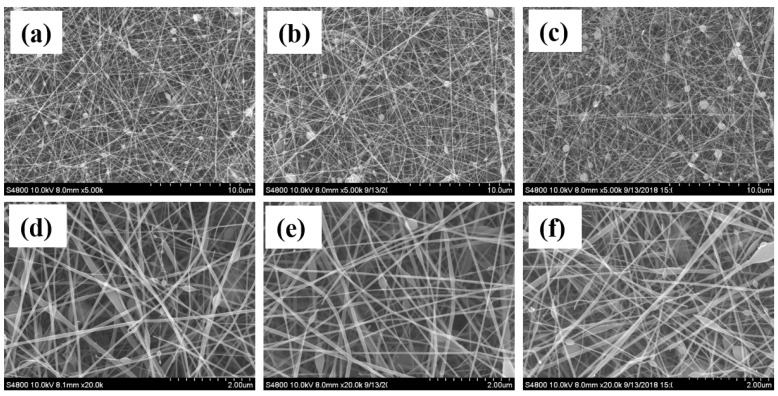
SEM images of SF nanofibers prepared by different electrospinning flow rates, at (**a**) 0.1 mL/h, (**b**) 0.15 mL/h, (**c**) 0.2 mL/h, (**d**) 0.1 mL/h, (**e**) 0.15 mL/h, (**f**) 0.2 mL/h. Note: Scale bars for (**a**–**c**) are 10 µm. Scale bars for (**d**–**f**) are 2 µm. Electric field: 1.2 kV/cm. Needle gauge: 20. SF concentration: 10 wt.%.

**Figure 2:**
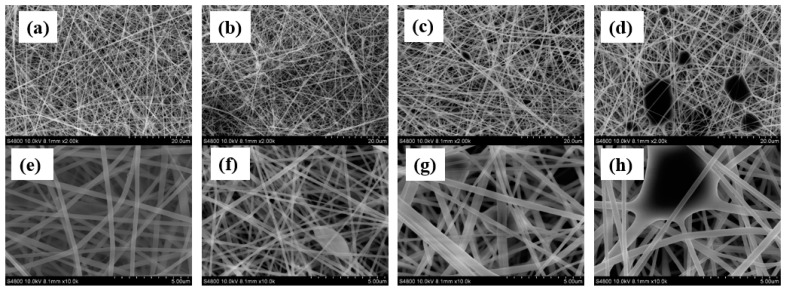
SEM images of SF nanofibers prepared by different electrospinning flow rates, at (**a**) 0.5 mL/h, (**b**) 0.6 mL/h, (**c**) 0.7 mL/h, (**d**) 0.8 mL/h, (**e**) 0.5 mL/h, (**f**) 0.6 mL/h, (**g**) 0.7 mL/h, (**h**) 0.8 mL/h. Note: Scale bars for (**a**–**d**) are 20 µm. Scale bars for (**e**–**h**) are 5 µm. Electric field: 1.83 kV/cm. Needle gauge: 20. SF concentration: 10 wt.%.

Figure 1 shows that the morphology of the electrospun SF was nanofibrous at the feeding rate ranging from 0.1 mL/h to 0.2 mL/h by using electrospinning electric field of 1.2 kV/cm. However, there were bead defects present in the SF nanofibers and also higher flow rate, leading to more beaded fibers, which was caused by the unavailability of proper drying time for electrospun fibers prior to reaching the collector [24]. According to the studies reported in literature, a higher electric field leads to greater stretching of the electrospinning solution and also rapid evaporation of solvent, so that the beaded morphology could be improved [24]. Therefore, a higher electric field of 1.5 kV/cm was applied to improve the bead defects in the resulting nanofibers. Figure S4 shows there are almost no beads present in the resulting SF nanofibers at the feeding rate ranging from 0.1 mL/h to 0.2 mL/h. Nevertheless, the electrospinning process was too slow, due to low solution feeding rate, in spite of success of forming nanofibers without any bead defects. Therefore, the feeding rate ranging from 0.5 to 0.8 mL/h was tested. Figure S5 shows the morphology of the SF nanofibers electrospun using an electric field of 1.66 kV/cm. It can be observed that there were still bead defects present in the resulting SF nanofibers and a higher flow rate resulted in more beaded fibers. Again, a higher electric field, of 1.83 kV/cm, was utilized to improve the bead defects in the resulting nanofibers. Figure 2 shows that feeding rate of 0.5 mL/h could be used to prepare SF nanofibers without any bead defects, although the beaded morphology appeared at the feeding rate greater than 0.5 mL/h.

**Figure 3:**
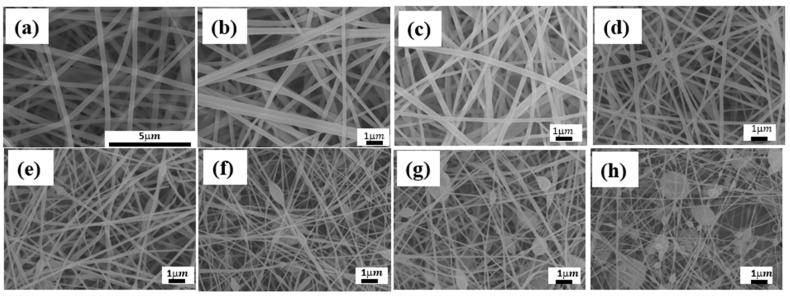
SEM images of SF nanofibers with different added amounts of PANI: (**a**) 0%, (**b**) 2.5%, (**c**) 5%, (**d**) 10%, (**e**) 15%, (**f**) 20%, (**g**) 25%, (**h**) 30%.

**Figure 4:**
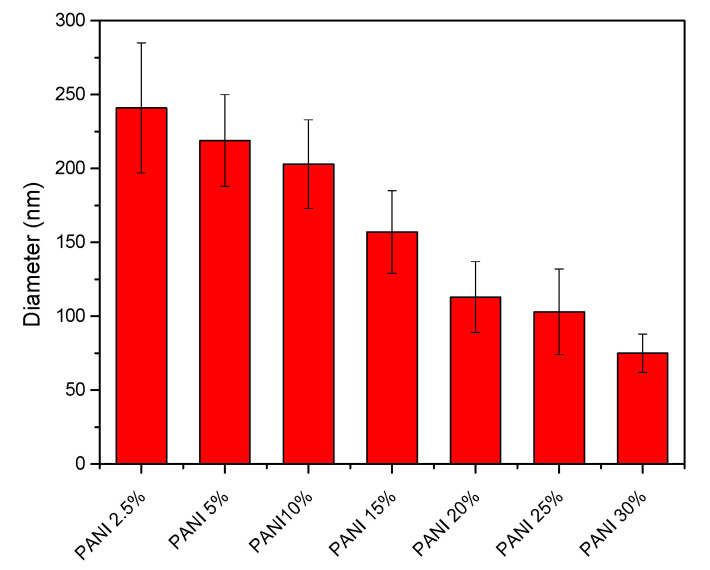
Effect of adding polyaniline (PANI) on the fiber size of the electrospun PANI/ SF nanofibers.

Accordingly, the optimized condition for electrospinning PANI/SF nanofibers was chosen as electric field of 1.83 kV/cm and feeding rate of 0.5 mL/h. The resulting PANI/SF nanofibers incorporating up to 15 wt.% of PANI were fibrous without any beaded morphology, as shown in Figure 3b–e. However, beads appeared as the added amount of PANI increased to 20 wt.% (Figure 3f) and adding more PANI in SF led to thinner fibers with more severe beaded morphology, as shown in Figure 3g,h. In addition to beading, introducing PANI also led to composite fiber with smaller diameters and the resulting electrospun mats with lower porosity. The fiber size of the electrospun PANI/SF composite nanofibers’ mats was evaluated and the results are shown in Figure 4. The diameter of the nanofiber was 241 ± 44 nm when adding 2.5% of PANI. The size continually decreased, to 75 ± 13 nm adding 30% of PANI. The porosity (ε) of the electrospun PANI/silk composite nanofibers’ mats is summarized in Table 2. The porosity decreased from 75% to 55% when increasing the added amount of PANI from 2.5% to 30%.

### 3.2. Properties of Electrospun PANI/Silk Composite Mats

**Figure 5:**
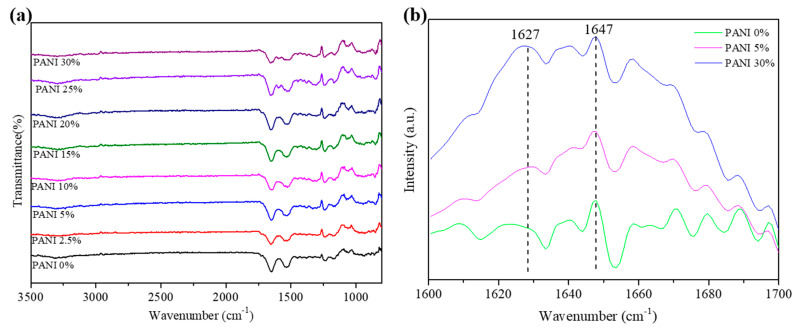
(**a**) FTIR spectra of electrospun PANI/silk mats with various amounts of PANI. (**b**) FTIR spectra of the amide I absorption band.

FTIR is a useful tool to determine not only the chemical structure but also the conformational structure of the protein-based materials. As seen in FTIR spectra in Figure 5a, all the prepared electrospun PANI/silk mats showed similar results. The peaks found close to 3000 cm^−1^ were due to the C-H stretching from silk fibroin and PANI. Furthermore, three major bands from silk fibroin, including amid I (C=O stretch, 1600–1700 cm^−1^), amid II (N-H deformation, 1500–1600 cm^−1^), and amid III (C-N stretch and N-H bending, 1200–1300 cm^−1^), also appeared [25,26]. Among these three amide modes, the amide I band is commonly utilized to determine the secondary conformational structure of silk fibroin. In general, the β-sheet crystalline conformation has a strong absorption band at 1620–1640 cm^−1^ and the random coil structure is normally found at 1640–1650 cm^−1^ [26]. Accordingly, wavenumbers of 1647 and 1627 cm^−1^, which belong to random coil and β-sheet crystalline structure, respectively, were chosen to investigate the effect of adding PANIs on the silk structure. Figure 5b shows that there was no appreciable peak appear at 1627 cm^−1^ for silk fibroin without adding any PANI, indicating the prepared electrospun silk nanofibers with few well-defined crystalline structures. However, the peak of 1627 cm^−1^ appeared when adding 5% of PANI in the silk fibroin. Furthermore, β-sheet crystalline conformation (1620–1640 cm^−1^) dominated compared to random coil structures (1640–1650 cm^−1^) when the added amount of PANI increased to 30%. These results suggest that adding PANI could induce the formation of β-sheet crystalline structure in the prepared electrospun silk nanofibers.

**Figure 6:**
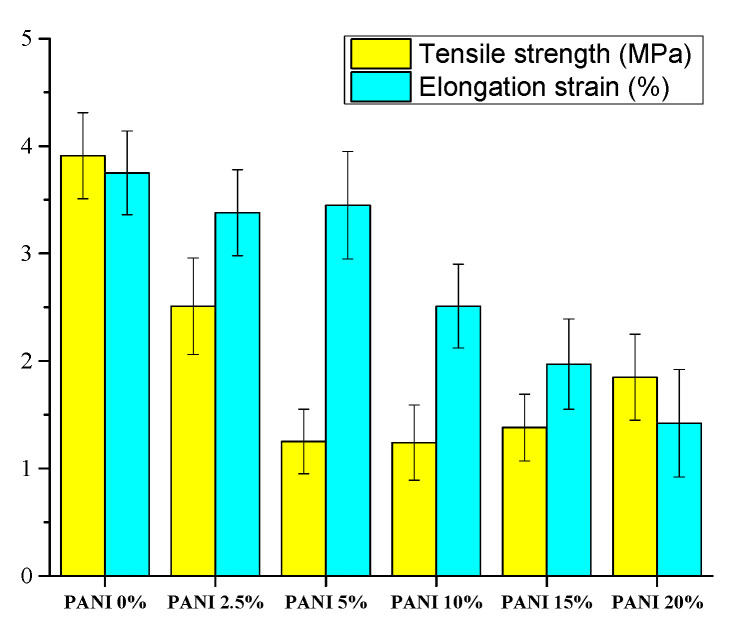
Effect of adding PANI on the mechanical properties of the electrospun PANI/ SF mats.

The mechanical properties of the resulting PANI/silk composite nanofibers’ mats are shown in Figure 6. The pure silk electrospun mats had tensile strength of 3.91 MPa with elongation strain of 3.75%. The tensile strength decreased to 2.5 MPa and 1.25 MPa when adding 2.5 wt.% and 5 wt.% of PANI. These results contribute that the molecular weight of PANI is less than silk so that more PANI led to lower tensile strength. However, the tensile strength maintained close to 1.3 MPa as the added amount increased to 15%. Moreover, the tensile strength increased to 1.8 MPa, instead of continuing to decrease, for the composite mat with 20% of PANI. These results are because the beaded morphology in the electrospun composite fibers (Figure 3e,f). Porosity measurement shows that more PANI led to low porosity, which indicated more compact composite mats. Therefore, composite mats with higher amount of PANI could have higher tensile strength. In contrast, the elongation strain of composite mats decreased when increasing the added amount of PANI. The reason is because PANI could be considered as a rigid polymer since its glass transition temperature is higher than 200 °C [27]. Therefore, rigid composite mats were obtained when introducing more PANI, so that its elongation strain decreased.

**Figure 7:**
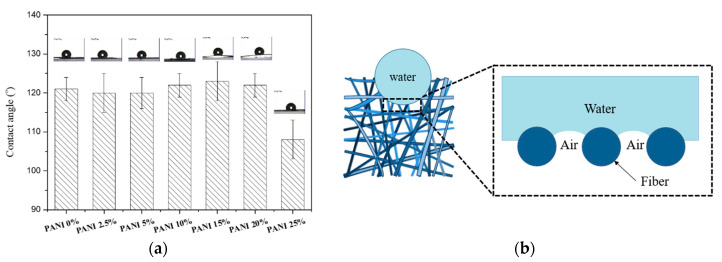
(**a**) Effect of adding PANI on the water contact angle of the electrospun PANI/ SF mats. (**b**) Illustration of silk fibroin/PANI composites’ morphology.

The textiles’ surface showing hydrophobicity is desirable for preventing fabrics from wetting. Thus, the water contact angle test was utilized to reveal the water wettability of the prepared PANI/silk composite nanofibers’ mats. Both silk fibroin and PANI are considered as hydrophilic polymer since their surface energy is close to 50 mJ m^−2^ [28,29]. However, the water contact angle of our electrospun silk mats was larger than 90° (Figure 7a), indicating the hydrophobic surface. The improved hydrophobicity of the silk fibroin/PANI composites was due to the entrapped air within the porous structures, as illustrated in Figure 7b. The water droplet partially wets the side surfaces of the composite fibers and partially sits on hydrophobic air pockets [30]. The resulting water contact angle was maintained close to 120° for composite mats with up to 20% PANI. However, water contact angle decreased to 108° when adding 25% PANI, due to the beaded morphology. The water wetting properties of porous textiles’ structures largely depend on surface roughness, pore size, and interpore spacing [31]. The mats with 25% PANI showed lowest porosity so that the water droplet can impregnate into mats more easily due to fewer air pockets on the surface, leading to smaller water contact angle compared to ones with less than 25% PANI.

### 3.3. Electrochemical Properties of Electroactive PANI/Silk Nanofibers

**Figure 8:**
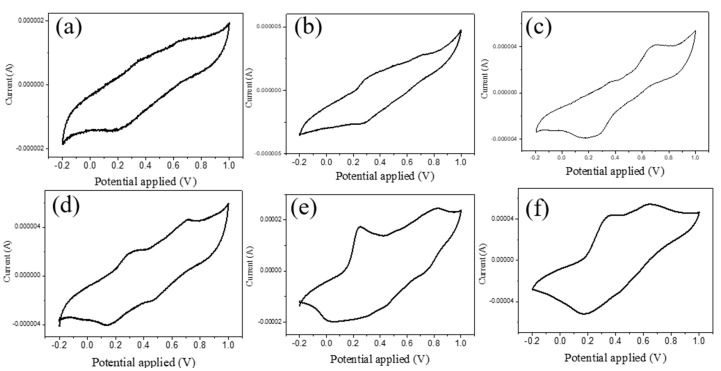
Cyclic voltammetry measurement for SF nanofibers with different added amounts of PANI: (**a**) 2.5%, (**b**) 5%, (**c**) 10%, (**d**) 15%, (**e**) 20%, (**f**) 25%.

**Figure 9:**
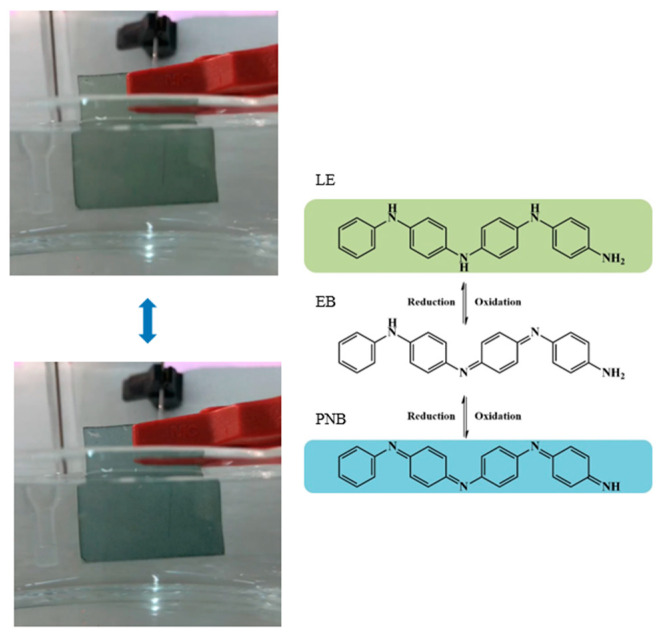
Effect of applied voltage on the color of PANI/SF mats. (Top: 0.2 V; bottom: 0.6 V).

The electrochemical-responsive behavior of PANI/silk nanofiber mats was investigated in 1.0 M sulfuric acid solution. There were distinct redox peaks present in the cyclic voltammetry test for all composite nanofibers with PANI, as shown in Figure 8. The first pair of redox peaks close to 0.3 V oxidation peak were due to the PANI’s transition from its fully reduced LE state to half-oxidized EB state. The second pair of redox peaks with the oxidation potential around 0.6 V were attributed to the transition from its half-oxidized EB state to fully oxidized PNB state. It is worth mentioning that PANI/silk composites need slightly higher applied potential for changing from EB to PNB state compared to the needed applied potential of 0.55 V for plain PANI. This is because PANI/silk nanofibers have lower conductivity, compared to plain PANI, so that they need higher voltage to initiate the redox reaction [32]. Furthermore, the redox current increased when increasing the PANI added amount to 25 wt.%. In addition, the applied voltage could change the color of the PANI/SF mats reversibly. Figure 9 shows that the composite mats exhibited as bluish and greenish as the applied voltage was 0.6 V and 0.2 V, relating to the PANI at PNB and LE state, respectively. These results confirm that PANI could enable the composite nanofibers to have electroactive and electrochromic properties.

**Figure 10:**
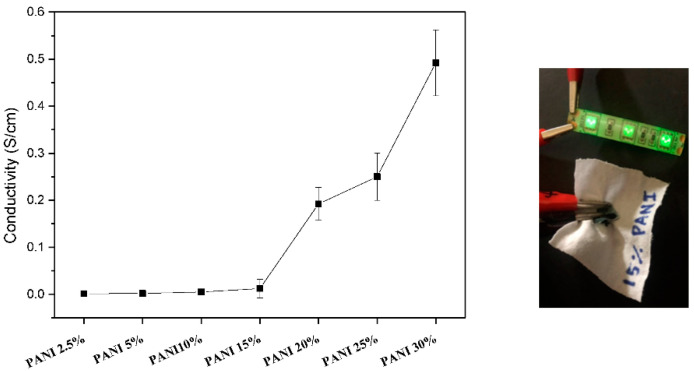
Electrical conductivity for SF composite mats with different added amounts of PANI.

The electrical conductivity of the PANI/silk nanofiber mats is shown in Figure 10. The silk composites exhibited high electrical resistance at low, incorporated PANI amount. In contrast, the electrical conductivity increased when increasing the incorporated amount of PANI and reached 0.5 S/cm when adding 30 wt.% PANI in the composite nanofiber. Nevertheless, the developed PANI/silk nanofiber mats could be sewn onto cotton fabric and then conduct electrical current to light up LEDs, as demonstrated in Figure 10.

## 4. Conclusions

We demonstrated the feasibility of developing highly conductive silk fibroin electrochromic nanofibers for use in E-textiles using electrospinning technique. PANI conductive polymer was synthesized and then incorporated to silk fibroin to fabricate electrospun silk composite nanofibers with high electrical conductivity and electroactive property. The spinning dope composition and spinning conditions, including solution feeding rate and electric field, significantly affected the morphology of the composite nanofibers. Thus, we could fine-tune the electrospinning procedure to control the morphology of the composite nanofibers, thus altering their mechanical properties and surface wettability. The developed composite nanofiber mats not only can conduct electrical current but also exhibit electroactive/electrochromic behaviors, which, by adjusting the applied voltage, could change their colors. Thus, the PANI/silk composite nanofibers developed in this study are promising for applications in E-textiles with multifunctionality.

## Figures and Tables

**Table 1:** Solution compositions for electrospinning.

PANI (%)	0%	2.5%	5%	10%	15%	20%	25%	30%
**Formic Acid (g)**	9	9	9	9	9	9	9	9
**Silk Fibroin (g)**	1	0.975	0.95	0.9	0.85	0.8	0.75	0.7
**PANI (g)**	0	0.025	0.05	0.1	0.15	0.2	0.25	0.3

**Table 2:** The porosity of the electrospun PANI/SF composite nanofibers’ mats with various amounts of PANI.

	PANI 2.5%	PANI 5%	PANI 10%	PANI 15%	PANI 20%	PANI 25%	PANI 30%
Porosity	75 ± 7.1%	74 ± 8.3%	73 ± 6.2%	71 ± 6.8%	67 ± 5.9%	64 ± 6.4%	55 ± 7.5%

## Supplementary Materials

The following are available online at https://www.mdpi.com/2073-4360/12/9/2102/s1. Figure S1. (a) Molecular structures of PANI with different redox states. (b) FTIR spectroscopy spectrum of PANI. Figure S2. UV-VIS spectra for (a) PANI in EB state, (b) PANI in LE state, (c) monitoring the chemical oxidation of the PANI in the LE state. Figure S3. Cyclic voltammetry measurement for synthesized PANI. Figure S4. SEM images of SF nanofibers prepared by different electrospinning flow rates, at (a) 0.1 mL/h, (b) 0.15 mL/h, (c) 0.2 mL/h, (d) 0.1 mL/h, (e) 0.15 mL/h, (f) 0.2 mL/h. Figure S5. SEM images of SF nanofibers prepared by different electrospinning flow rates, at (a) 0.5 mL/h, (b) 0.6 mL/h, (c) 0.7 mL/h, (d) 0.8 mL/h, (e) 0.5 mL/h, (f) 0.6 mL/h, (g) 0.7 mL/h, (h) 0.8 mL/h.
