# Supplementary material for: Electrospun Hydrophobic Polyaniline/Silk Fibroin Electrochromic Nanofibers with Low Electrical Resistance
Source: Polymers (Basel). 2020 Sep 16;12(9):2102. doi: 10.3390/polym12092102 (PMC7569898; doi:10.3390/polym12092102)
Supplement: Supplementary file 1 [file polymers-12-02102-s001.pdf]

polymers

Article

Electrospun Hydrophobic Polyaniline/Silk Fibroin Electrochromic Nanofibers with Low Electrical Resistance

Chun-Yu Chen[1], Szu Ying Huang[1], Hung-Yu Wan[1], Yi-Ting Chen[1], Sheng-Ka Yu[1], Hsuan-Chen Wu[2*] and Ta-I Yang[1*]

[1] Department of Chemical Engineering, Chung-Yuan Christian University, Taoyuan, Taiwan
[2] Department of Biochemical Science and Technology, National Taiwan University, Taipei, Taiwan
* Correspondence: Corresponding authortaiyang@cycu.edu.tw (T. –I. Yang) Tel.:+886-3-2654149; fax: +886-3-2654199; hcwu7@ntu.edu.tw (H.-C. Wu) Tel.: +886-2-33664524.

Received: 4 August 2020; Accepted: 10 September 2020; Published: 16 September 2020

Supplementary materials

There are three distinguishable oxidation states in PANI, which are fully reduced (leucoemeraldine base) (LEB), the half oxidized (emeraldine base) (EB), and the fully oxidized (pernigraniline base) (PNB) states as illustrated in Figure S1(a)[1].The chemical structure synthesized PANI was analyzed by FTIR (Figure S1(b)). The peak of 1504 cm^{-1} and 1594 cm^{-1} could be assigned to benzenoid and quinoid rings stretching vibrations, respectively. There three peaks at 1309, 1164 and 834 cm^{-1} is from the C–N stretching vibration of a secondary aromatic amine, the aromatic C–H in-plane deformation, and the C–H out-of-plane deformation of 1,4-aromatic substituted benzene rings, respectively. The broad peaks close to 3314 cm^{-1} and 3208 cm^{-1} could be assigned to H-bonded N-H stretching [2].

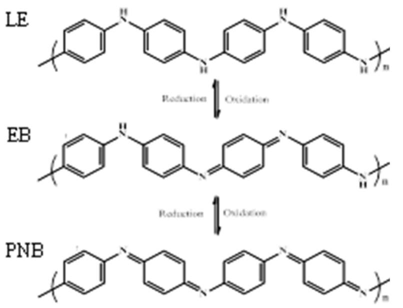

(**a**) (**b**)

Figure S1. (a) Molecular structures of PANI with different redox states, (b) FTIR spectroscopy spectrum of PANI.

Optical properties of as-prepared PANI were obtained at room temperature using a UV-visible spectroscopy. The as-prepared emeraldine base of PANI showed two distinct absorption bands at 324 nm and 624nm, which are attributed to the π -π^* transition in the benzenoid ring and exciton absorption in the quinoid rings, respectively (Figure S2(a))[3, 4]. Moreover, hydrazine could completely transformed as-prepared PANI from EB form to leucoemeraldine (LE) form so that there is only one absorption band present at 336 nm (Figure S2(b)), which associating with the π -π^*

transition of the benzenoid rings in LE form of PANI. The reduced LE PANI could be oxidized by adding ammonium persulfate oxidant. Figure S2(c) of UV-vis spectra shows that he intensity of the bands close to 320 nm decrease because the benzenoid rings transformed to quinoid rings as the added amount increased. At the same time, the intensity of the 620 nm band, associating with exciton absorption in the quinoid rings, increased due to the ratio of quinoid rings in PANI increased.

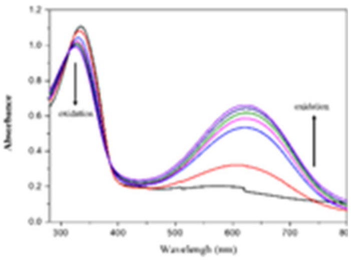

(**a**) (**b**) (**c**)

Figure S2. UV–vis spectra for (a) PANI in EB state, (b) PANI in LE state, (c) monitoring the chemical oxidation of the PANI in the LE state.

The CV tests for the synthesized PANI in Figure S3 show that there are two oxidation peaks at 0.31V and 0.55 V vs. Ag/AgCl, which are attributed to the transition from fully reduced LE state to half oxidized EB state and half oxidized EB state to fully oxidized PNB state, respectively. The result shows electroactive PANI have been successfully synthesized.

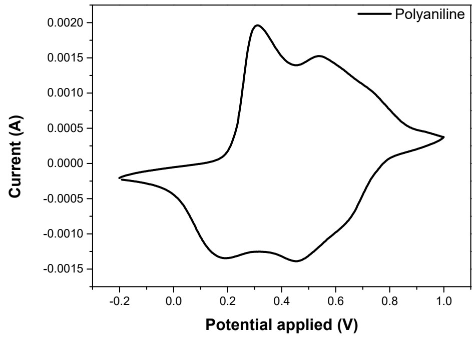

Figure S3. Cyclic voltammetry measurement for synthesized PANI.

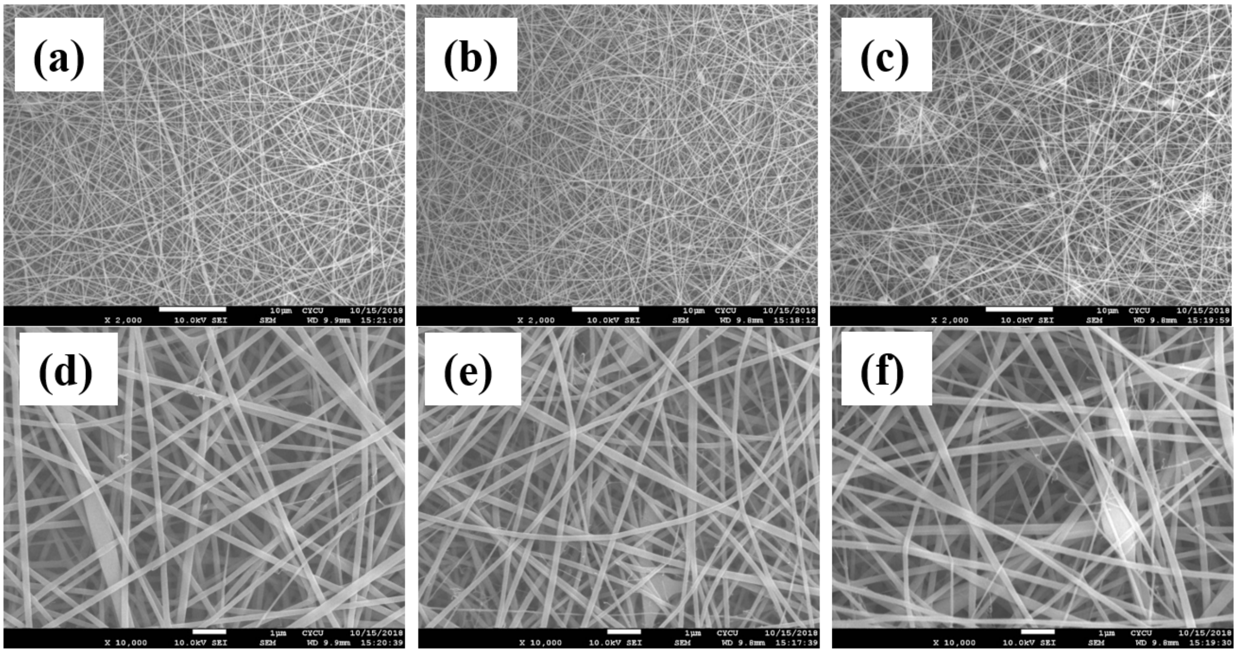

Figure S4. SEM images of SF nanofibers prepared by different electrospinning flow rate. (a) 0.1 ml/hr, (b) 0.15 ml/hr, (c) 0.2 ml/hr, (d) 0.1 ml/hr, (e) 0.15 ml/hr, (f) 0.2 ml/hr.

Note: Scale bars for (a), (b), and (c) are 10μm. Scale bars for (d), (e), and (f) are 1 μm. Electric field: 1.5 kV/cm. Needle gauge:20. SF concentration: 10 wt%.

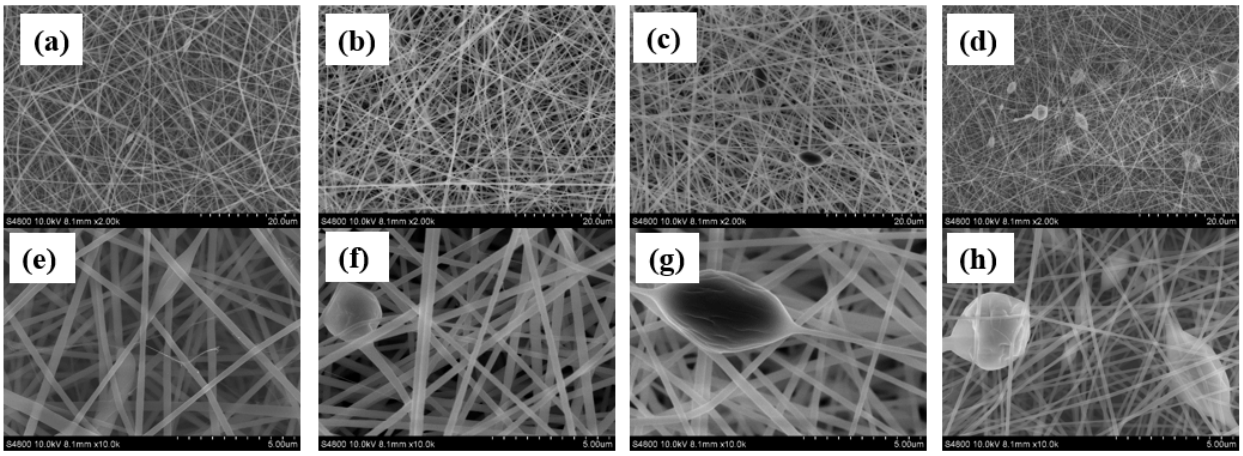

Figure S5. SEM images of SF nanofibers prepared by different electrospinning flow rate. (a) 0.5 ml/hr, (b) 0.6 ml/hr, (c) 0.7 ml/hr, (d) 0.8 ml/hr, (e) 0.5 ml/hr, (f) 0.6 ml/hr, (g) 0.7 ml/hr, (h) 0.8 ml/hr.

Note: Scale bars for (a), (b), (c), and (d) are 20μm. Scale bars for (e), (f), (g), and (h) are 5 μm. Electric field: 1.66 kV/cm. Needle gauge:20. SF concentration: 10 wt%.

Reference

1. Baker, C.O., et al., *Polyaniline nanofibers: broadening applications for conducting polymers.* Chemical Society Reviews, 2017. **46**(5): p. 1510-1525.
2. Trchová, M. and J. Stejskal, *Polyaniline: The infrared spectroscopy of conducting polymer nanotubes (IUPAC Technical Report)*, in *Pure and Applied Chemistry*. 2011. p. 1803.
3. Huang, W.S. and A.G. MacDiarmid, *Optical properties of polyaniline.* Polymer, 1993. **34**(9): p. 1833-1845.

4. Nekrasov, A.A., V.F. Ivanov, and A.V. Vannikov, *Analysis of the structure of polyaniline absorption spectra based on spectroelectrochemical data.* Journal of Electroanalytical Chemistry, 2000. **482**(1): p. 11-17.

© 2020 by the authors. Licensee MDPI, Basel, Switzerland. This article is an open access article distributed under the terms and conditions of the Creative Commons Attribution (CC BY) license (http://creativecommons.org/licenses/by/4.0/).
